# A forum on innovative fusion approaches: will there be a SpaceX for fusion energy?

**DOI:** 10.1093/nsr/nwz098

**Published:** 2019-07-22

**Authors:** Weijie Zhao

**Affiliations:** NSR news editor based in Beijing

Among all the clean-energy approaches, fusion energy is generally considered to be the best option: fusion fuels such as deuterium (D) can be extracted from sea water while lithium for breeding tritium (T) is an abundant mineral and almost infinite in reserve; the fusion reaction is completely carbon-emission-free and generates no waste except for a small and manageable amount of radioactivity; fusion power plants are failsafe, free of potential nuclear disasters. However, fusion reaction occurs at extremely high temperature and pressure, so it is not easy to reach the ignition point and to have a container that confines the reaction. People have been pursuing fusion energy for more than six decades, but no fusion power plants have been built.

Magnetic confinement fusion (MCF) represented by tokamaks is presently the most mature experimental approach. The international fusion energy project ITER (‘The Way’ in Latin) is building the world’s largest tokamak and plans to start D-T operation by the end of 2035 and achieve fusion power (500 MW) equal to ten times the plasma-heating and current-drive power (50 MW for a Q=10) starting in 2038. Note that the Q value cited here is not relative to the total power draw from the grid, which will be substantially more than the 50 MW delivered to the plasma. Besides MCF, inertial confinement fusion (ICF) has also made significant progress in recent years and is considered to be another mainstream approach.

In addition, there are also innovative/alternative approaches that are less mature and more risky. However, some researchers believe that these innovative approaches could yield smaller and cheaper fusion power plants within the next couple of decades, raising the interest and ambition of private enterprises in entering this field. In May 2019, the First International Conference on Innovative Fusion Approaches was held in Xi’an, China. *National Science Review* held a panel discussion on the future opportunities and challenges of the innovative approaches in fusion energy. The following contains excerpts of the panel discussion.


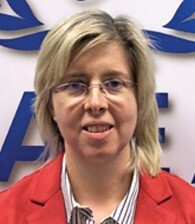


**Sehila M. Gonzalez de Vicente**

Nuclear Fusion Physicist at the International Atomic Energy Agency, Austria


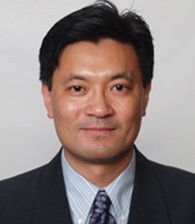


**Houyang Guo**

Director of the DIII-D Plasma Materials Interaction Center of General Atomics, USA


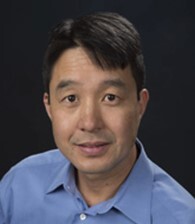


**Scott Hsu**

Program Director at the Advanced Research Projects Agency–Energy, Department of Energy, USA


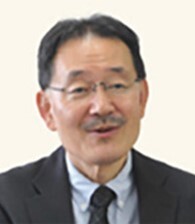


**Shigeo Kawata**

Professor at Graduate School of Regional Development and Creativity, Utsunomiya University, Japan


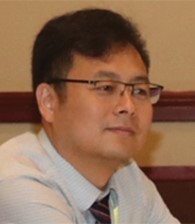


**Yongtao Zhao**

Professor at School of Science, Xi’an Jiaotong University, China


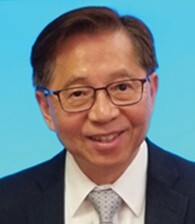


**Yong Chia Francis Thio (Chair)**

CEO and Chief Scientist of HyperJet Fusion Corporation, USA; now at Breakthrough Fusion Corporation, USA


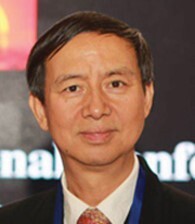


**Xianjun Yang (Chair)**

Professor at Institute of Applied Physics and Computational Mathematics, China

## Technical, economic and political challenges

**Yang:** The concept of fusion energy was proposed more than 65 years ago, but has not yet been realized. In your opinion, what are the major difficulties?

**Hsu:** I think there are two major considerations—one being economic and the other scientific and technical. Most of the world is now focusing on the scientific and technical considerations, but I think we need to focus more on the economic and market considerations, which can make fusion more relevant to the real world.

I work in the Advanced Research Projects Agency–Energy (ARPA-E) of the US Department of Energy (DOE), which funds diverse energy approaches. So I understand that fusion is not the only way to solve the energy crisis, as there are a number of viable options out there such as carbon sequestration, long-duration energy storage, geothermal energy and even taking carbon out of the atmosphere.

Right now it is a ‘motherhood’ statement that fusion energy is the holy grail of energy and that we need it in the long run; but we are unable to provide the current energy industry any details on how to get there. We cannot yet see how fusion energy will integrate into the current energy system and what the things are that fusion energy can provide that other energy sources cannot. In the USA, fusion energy has been particularly challenging because it does not match the market conditions.

We should clarify the market requirements, and then go back to science and technology (S&T) research to look for the approaches that have a potential to satisfy these requirements. We need to consider more concepts that are smaller, less complex and with lower cost.

**Gonzalez:** Fusion may not fit into the market right now. But in the last 15 years, many fusion companies were set up and many fusion facilities were built throughout the world. I think fusion is entering a new era, moving from the scientific stage to the realization stage. The final goal is to build a commercially competitive fusion power plant.

When I joined the International Atomic Energy Agency (IAEA) four and a half years ago, there were few people interested in fusion energy. But now things have changed completely. Investors noticed this field and private enterprises are going up. We began to talk about the costs and other realistic issues about the future fusion power plant.

Fusion has been an international attempt from the very beginning. For fission, every single country has its own design. But there is no single country able to construct a fusion machine all by itself. IAEA would like to be the platform to put people together for the common final goal.

Another major challenge is how to get more support from the policy makers. —Xianjun Yang

**Yang:** I think another major challenge is how to get more support from the policy makers. If the policy makers can put more emphasis on fusion energy, especially on the innovative approaches which may give rise to a fusion power plant within 20 or 30 years, things would be changed.

**Gonzalez:** Political supports, including budget, policy and talent are actually important. In recent years, the policy makers are very concerned about the greenhouse effect and climate change. So in the first page of a proposal, we should clearly describe how fusion would help to mitigate the effect of climate change. This would greatly help us to get more funds.

**Kawata:** That is right. We know that fusion energy is emission-free and is promising, but the politicians and the public do not know much about it. We should try to increase the public acceptance of fusion energy. And on the other hand, if one day we are able to build a fusion power plant and the plant is not completely radioactive-free, this knowledge should also be honestly announced to the public.

**Thio:** There are also fundamental technical challenges for the development of practical and efficient fusion power plants. The formulation of any fusion approach intended to be a practical fusion power plant should begin by addressing these fundamental issues. One of them would be the problem with neutrons. If your approach uses D and T as fuels, the fusion reaction would generate neutrons and you need to deal with them. One way to deal with the neutrons is to use a thick liquid wall as the first wall.

**Figure:**
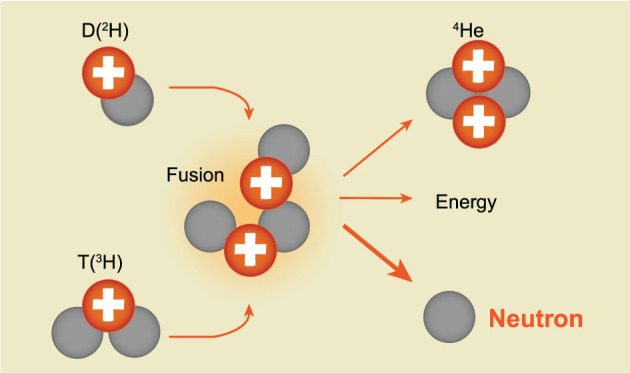
D+T fusion reaction generates neutrons.

**Hsu:** It’s worth pointing out that every private fusion company either utilizes a thick liquid wall or uses advanced fuels. The upfront physics for advanced fuels is not very easy, but once it is solved, the following engineering will be easier. This is an advantage from the economic point of view.

## Magneto-inertial fusion (MIF): a hybrid of MCF and ICF

**Guo:** MIF is an innovative fusion approach that combines many desirable features of both MCF and ICF. MIF is currently not as mature as the traditional approaches such as tokamaks, but I think there is potential for this concept to be transformative

The physics became very difficult and complicated when we try to combine the two traditional approaches.—Yong Chia Francis Thio

for fusion energy development and it deserves an opportunity to be further explored.

**Hsu:** Theoretically, MIF requires less stored energy and less heating power to get to a thermonuclear condition. This idea has been tested and has achieved good results. An example is the Z pulsed-power machine constructed by the DOE’s Sandia National Laboratories.

MIF is not as mature as tokamaks. It is hard for people to imagine that something less mature is going to realize fusion energy in a shorter time. But if you look at the history of such scientific achievements, you can see that we often overestimate what we can do in the short term but underestimate what we can do in the long term because we cannot imagine the breakthroughs that are going to happen. So I think if we can provide an environment for breakthroughs to occur, it is entirely possible that a fusion concept that is currently less mature will leapfrog a mature approach and generate the ultimate first fusion power plant.

**Kawata:** I essentially agree with you. But in order to reach the final goal, we have to solve the problems of instability and repetition rate.

**Thio:** That is right. These two problems attracted my attention in the mid-1990s and led to the formulation of the concept of Plasma Jet Driven Magneto-Inertial Fusion (PJMIF), which could be one of the potential solutions.

For solid-liner-driven MIF, the destruction of the liner and the transmission lines make it difficult to deal with the issue of repetition rate. In PJMIF, an array of high-velocity dense plasma jets is used to deliver the momentum flux required to compress the target plasma to ignition from a standoff distance at high implosion velocity without destroying any hardware, thus potentially enabling high repetition rate. However, PJMIF is at a very early stage. We do not have those kinds of jets yet.

MIF is actually a very broad class of fusion approaches with a very large parameter space. There are a number of embodiments of MIF, which use solid liners, liquid liners or plasma liners. And for each kind of liner, there are several ways to realize it.

MIF is a hybrid of magnetic fusion and inertial fusion. But the combination is not easy. The physics became very difficult and complicated when we try to combine the two traditional approaches.

**Hsu:** You get the advantages of both, but you also get the challenges of both.

**Thio:** Exactly!

**Yang:** I think MIF is a leading example of the innovative fusion approaches. It offers a lot of choices to reach the final practical goal. I think it is the right time to rethink about our past fusion attempts. It is very likely that these new concepts will speed up our pursuit of fusion energy.

## Multiple innovative fusion approaches

**Yang:** What are the other innovative approaches except for MIF?

**Zhao:** We are working on heavy-ion inertial fusion, which is also a competitive candidate for fusion energy. The energy-conversion efficiency of heavy-ion fusion is very high, much higher than laser fusion and other approaches.

Another point is that we can combine heavy-ion fusion with other approaches such as MIF or Z pinch. For example, we can use heavy ions to produce high-density, high-temperature plasmas and then use other approaches for ignition, or vice versa.

Besides, we should also pay attention to the new phenomena for the matter and radiation at extremes, where non-linear effects may take place. Recently, we did some experiments using laser-accelerated ion beams. The beam density is extremely high and the energy deposition in materials is more than one order higher than the existing theoretical predicts. In this case, we need change the view of alpha heating, ion fast ignition and heavy inertial fusion, even the scheme of ICF.

So I think we can combine different approaches and we should have an eye to something new.

**Thio:** There has also been progress in gas-dynamic traps. Within the last five years or so, this concept reached 1-keV electron temperature, which can be considered as a major threshold for fusion research. When the Russian T3 tokamak achieved 1-keV temperature in the 1960s, the whole world dropped everything else and focused on tokamaks.

**Gonzalez:** Some Russian researchers are working on magnetic mirrors. Their works are also very valuable.

**Guo:** Our goal is to build an economic fusion plant. Starting from this final goal, we can identify a number of very promising ‘alternative’ configurations to achieve the desired features.

For example, a few private enterprises are pursuing field-reversed configuration (FRC). The geometry of FRC is very simple and it may potentially address some key issues such as disruptions and heat exhausting. There are also other interesting configurations such as mirrors and spheromaks. These alternative configurations exhibit highly desirable engineering features for an economic fusion reactor, but their physics is not mature because they have not been given lots of funding and time to perform further research.

We should also pay attention to the new phenomena for the matter and radiation at extremes, where non-linear effects may take place. —Yongtao Zhao

## The current fusion-research landscape: MCF as the giant

**Thio:** FRC is probably the most funded and the most mature of all the innovative fusion approaches besides magnetic mirrors and heavy-ion fusion. The cumulative governmental funding for FRC is less than 1% of the total fusion budget. The US government spent over 20 billion dollars in MCF, and not more than 200 million in FRC. Even less funding (per concept) has been spent in the other innovative fusion approaches except perhaps magnetic mirrors or heavy-ion fusion.

A typical criticism of innovative fusion approaches is: Where is the result? Where is the data? Tokamaks accumulated a large amount of data, but the innovative approaches did not have the budget to get that data. And the limited budget for innovative fusion approaches has to be spread over many innovative concepts, which made the situation even worse.

**Hsu:** There are two key recommendations in the recently released US National Academies report. One is that ITER is still the lowest-scientific-risk fusion path, so the USA should continue to pursue it. But the second main recommendation is that we should pursue a compact fusion pilot plant at the lowest capital cost. I think the lowest capital cost mentioned here includes both the developmental cost and the final power-plant cost. This is a great opportunity for the innovative approaches and is a very profound departure from the present US strategy.

**Zhao:** There are not as many private fusion companies in China as in the USA. But the Chinese fusion companies such as the ENN Group are becoming active in this field.

Our university, Xi’an Jiaotong University, has just established the West China Science and Technology Innovation Harbor. We are planning to build an Innovative Fusion Research Center in the Innovation Harbor. We will cooperate with Professor Francis Thio and Professor Dieter Hoffmann to do something new and practical. One of our plans is to build a heavy-ion accelerator in MeV region, combining with a strong laser and some magnetized plasma devices to study the fundamental physics of diverse fusion approaches.

**Kawata:** The major fusion-research direction in Japan is also MCF. We have the Large Helical Device (LHD), which is the second largest superconducting stellarator in the world. Many universities work on magnetic fusion. But there are also researches on other approaches. Osaka University researchers work on laser fusion. Tokyo Institute of Technology (TIT), the High Energy Accelerator Research Organization (KEK) as well as several universities including my university work on

We have [Japan has] the Large Helical Device (LHD), which is the second largest superconducting stellarator in the world. —Shigeo Kawata

heavy-ion-fusion experiment and theory. But generally speaking, Japan’s input for fusion energy is not as much as the USA and some other countries.

## The high wall separating mainstream and innovative concepts

**Guo:** Within the magnetic-fusion effort, fusion energy has been pursued along two pathways featuring: (i) Advanced Magnetic Confinement, such as tokamaks; (ii) Simple Magnetic Topology, as exemplified by FRC. For the mainline tokamak approach, we can firstly build a big ITER-like fusion reactor, and then try to reduce the cost and make it more compact. And for the other concepts, we need to make great breakthroughs in physics. The challenge of the first route is largely driven by engineering to make the huge facility smaller, and the difficulty of the second route is the upfront physics problems. I think we should persue both of them.

**Gonzalez:** IAEA would like to support every single approach and facilitate the exchange of information. Any helpful approach, traditional or innovative, should be allowed to develop. It is not a fight or a competition. It is a striving towards a common goal.

I have to mention that the lack of communication between the traditional and innovative researchers is a key problem. Many private enterprises are quite ambitious, but they commonly do not want to share their ideas and progress with the mainline researchers. They seldom give presentations in forums or conferences.

Maybe they are afraid of receiving unreasonable criticisms from the mainline researchers. But communication is the only way to let other people understand what you are doing. Good communication can help to improve your ideas and also attract more funds for your project.

It is not a fight or a competition. It is a striving towards a common goal. —Sehila M. Gonzalez de Vicente

**Guo:** There is an intellectual property (IP) challenge here. The private companies do not want to share their new ideas because they want to hold their IP. So the result is a one-way communication: they learn about what the mainline researchers are doing, but do not share information from their own side. How to open their doors might be very challenging. We need to find a way to break this barrier.

## Take advantage of the non-fusion technologies

**Guo:** Recently, we have some new developments on the enabling technologies which can potentially contribute greatly to fusion energy. For example, theoretical predictive modeling and advanced machine learning have already been used in guiding fusion experiment and data analysis. Other technologies such as high-temperature superconductors and advanced manufacturing also become key factors for fusion development. These could potentially mitigate and transform some present physics challenges from possibly insurmountable to potentially achievable.

**Gonzalez:** That is right. There are a number of new technologies that are really useful for fusion. And I think the innovative fusion approaches can incorporate enabling technologies much easier because of their flexibility. New technologies such as machine learning, 3D printing and new materials can be easily integrated and tested in the new approaches.

And we should not ignore fission. The knowledge, technology and experiences of fission can provide us many useful hints.

At this stage fusion researchers have to look carefully around because solutions to our issues may be found in other research fields. Actually, superconductors, which are currently major parts of fusion facilities, were borrowed from other research fields.

**Hsu:** I agree completely. We cannot reinvent the wheel for everything. I also want to address the lessons from fission. There are dozens of advanced fission concepts which are completely feasible in theory but none of them were ever built. Why is that? Fusion has to think about it right now and avoid falling into the same situation.

We have some new developments on the enabling technologies which can potentially contribute greatly to fusion energy. —Houyang Guo

## How to set a roadmap for fusion energy?

**Gonzalez:** IAEA is now writing an International Technical Roadmap. This report will point out the existing gaps needed to be fulfilled for the construction of a future fusion power plant. We will invite five or six experts to write it, and then send it to more experts for feedbacks before its formal release. The reviewers will include experts of mainline fusion approaches, innovative fusion approaches as well as non-fusion fields.

**Thio:** During the last 20 years, we have gap studies (convened by the government) almost every three or four years in the USA. But they were soon put on the shelves. The challenge of doing a gap analysis is that if 90% of our community work on just one specific approach, the outcome of the gap analysis will be

A lot of private fusion companies are asking for programs to set the milestones. —Scott Hsu

an analysis of that certain approach and it will not be a good guidance for the development of the other fusion approaches. How would your report avoid these situations?

**Gonzalez:** Firstly, our report will not be a gap analysis for a certain approach. We will listen to the experts from different countries and different approaches.

Secondly, this report will not be a roadmap that tells the researchers what they should do. It is a report written for the policy makers and will help the researchers to get funds from the governments or the investors. We hope this report can thus help the fusion community.

**Guo:** If we want to set a real roadmap for fusion energy, I think we need to set timely goals. Without a timeline, the development can be very lengthy and it will be difficult to achieve fusion energy within any reasonable time.

**Hsu:** In fact, a lot of private fusion companies are asking for programs to set the milestones. They are calling for a reimbursement program similar to the Commercial Orbital Transportation Services (COTS) program of the National Aeronautics and Space Administration (NASA). In the COTS program, the US government ended up paying 800 million dollars to develop a low-Earth-orbit launch vehicle.

In such a reimbursement program, the government set several milestones. Every time a private company meets a milestone, they would get reimbursed with some amount of money**.** For the government, this is a low-risk program. If the companies do not meet the milestone, the government will not pay anything. And for the companies, they will do the research more effectively in order to save money. The Nuclear Innovation Alliance just released a report relevant to this, which is titled ‘Enabling Nuclear Innovation: In Search of a SpaceX for Nuclear Energy’.

If the talents of the scientific community and of the private enterprises can be brought together, I think we have a chance to solve commercially viable fusion energy in 20 years.

**Yang:** Thanks very much for sharing your opinions! I personally believe that the innovative approaches such as MIF are very promising and I hope that the public and the policy makers can learn more about fusion energy and offer more support to this field.

Weijie Zhao is an NSR news editor based in Beijing.

